# Design Principles for Air Tolerance in Pyridinium‐Based Flow Batteries

**DOI:** 10.1002/adma.202508875

**Published:** 2025-11-05

**Authors:** Mark E. Carrington, Erlendur Jónsson, Clare P. Grey

**Affiliations:** ^1^ Yusuf Hamied Department of Chemistry University of Cambridge Cambridge CB2 1EW UK

**Keywords:** air stability, long duration energy storage, redox flow batteries

## Abstract

Pyridinium compounds represent promising electrolyte candidates for aqueous redox flow batteries. Recently, their ability to afford air‐stability was demonstrated, unlocking potential avenues both for relaxed system constraints and for high voltage operation. Here, simple equilibrium models for pyridinium electrolytes are developed, which are leveraged to predict and successfully validate the air stability of methyl viologen – the lowest cost and most well‐studied pyridinium system to date. By controlling the degree of π‐association of active species, the total fraction of radicals can be kept below a critical threshold, from which air‐stable operation can be accessed. The resulting system exhibits 94.9% capacity retention in air after 150 cycles but undergoes dramatic losses in performance once diluted outside of its air stability threshold. We tie this behaviour to rates of oxygen consumption in solution and further derive the second Damköhler number, a dimensionless parameter which informs optimal scaling of battery components. On this basis, air stability is shown to be compatible with scaling requirements needed for applications in long‐duration energy storage. Given the known tendency for broader classes of organic electrolytes to associate, it is anticipated that the findings presented can be generalized to many other current and future systems.

## Graphical abstract

Equilibrium models are derived for redox flow battery electrolytes that enable accurate prediction of the onset air stability in methyl viologen – a compound known to the community for over 100 years and one of the most well‐studied aqueous flow battery electrolytes.

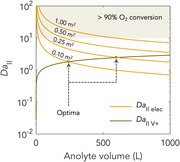

## 1 Introduction

Reduced pyridinium species exhibit a broad variety of discrete and assembled structures in solution.^[^ ^1^, ^2^, ^3^, ^4^ ^]^ These include radicals, π‐dimers,^[^ ^1^ ^]^ σ‐dimers,^[^ ^2^ ^]^ charge transfer complexes,^[^ ^2^ ^]^ higher order aggregates,^[^ ^3^ ^]^ and in some cases diradicals and their corresponding associated species^[^ ^4^ ^]^ – several of which may be present at a given state of charge (SOC). Among these species, π‐dimers were recently shown to afford both improved electrolyte stability and pathways through which air stability can be accessed in redox flow battery (RFB) applications.^[^ ^4^, ^5^ ^]^ This stability is thought to be achieved through an equilibrium between unpaired radicals and their corresponding π‐dimers (with associated equilibrium constant *K_d_*), which effectively lowers the fraction of unpaired radicals in the system as concentration is increased. The low fraction of open shell species in solution not only decreases reactivity toward oxygen but reduces the overall propensity for parasitic processes improving overall cycling stability. To date, these characteristics have enabled demonstrations of air stability in extended bispyridinium compounds.^[^ ^4^, ^5^ ^]^ They have also led to the emergence of useful physico‐chemical descriptors such as the dimerization constant (*K_d_*), from which preliminary assessments of air stability can be made.^[^ ^4^, ^5^ ^]^ However, a more general understanding of these characteristics and their applicability toward large‐scale application remains to be developed. Furthermore, a sufficient modelling framework of the dynamic equilibria of radical‐pairing‐based assembly of pyridinium compounds and how it can be utilized is lacking. Here, we present a simple but effective set of principles for assessing air‐stability in pyridinium‐based RFBs, which we use to successfully predict the air stability of methyl viologen – a canonical compound known to the community for over 100 years.

## 2 Generalised Equilibrium Models for Accurate Concentration Predictions at All SOCs

**Figure 1:**
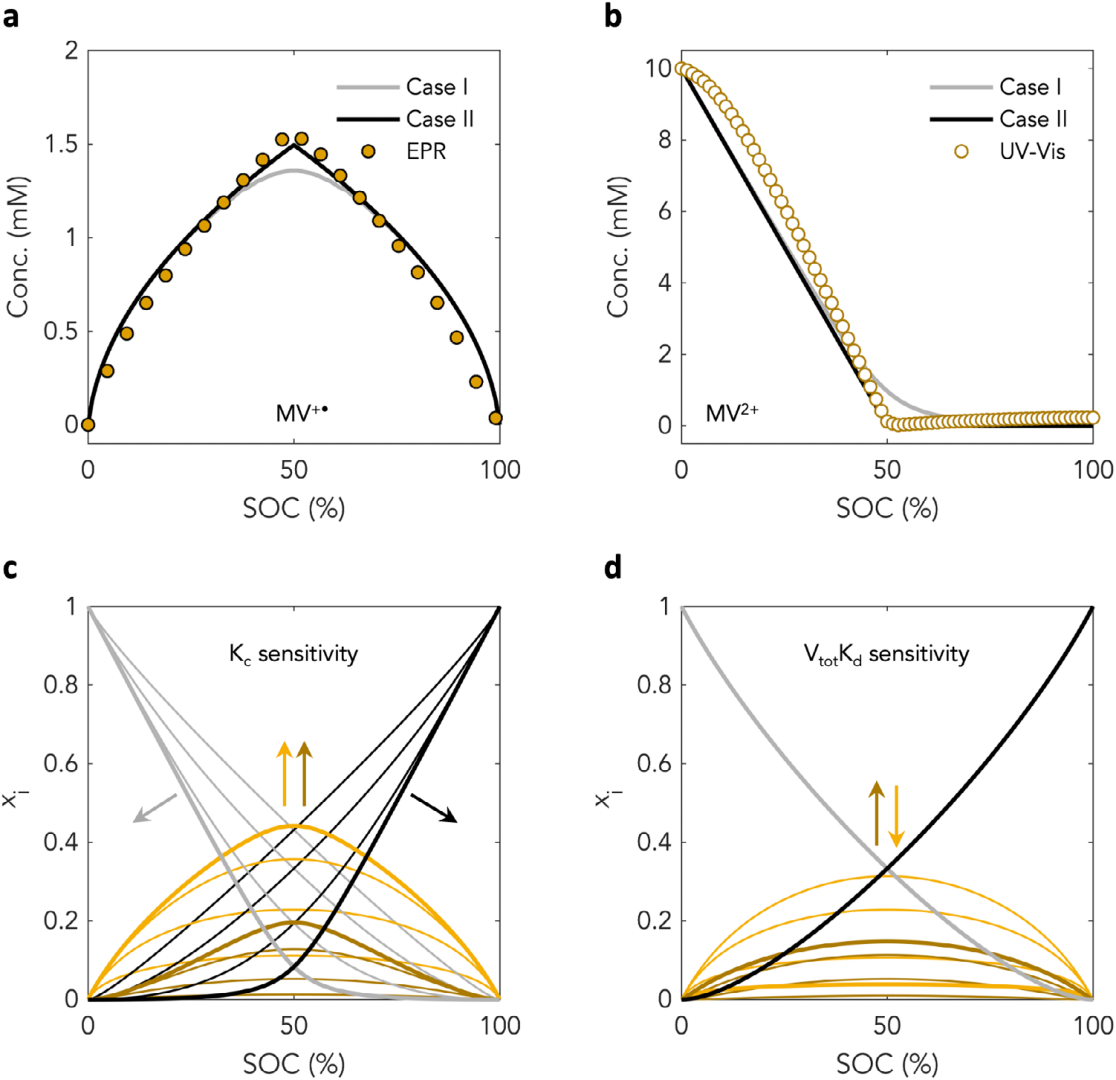
Models for a two‐electron system undergoing comproportionation and dimerization. a) ***Case I*** and ***Case II*** predictions for [MV^+•^_(aq)_] as a function of SOC compared with experimental data derived from EPR spectroscopy, where MV refers to methyl viologen.^[^ ^4^, ^5^ ^]^ b) ***Case I*** and ***Case II*** predictions for [MV^2+^_(aq)_] as a function of SOC compared with experimental data derived from UV–is spectroscopy (note that [MV^2+^_(aq)_] is diamagnetic so no EPR data are available. c) ***Case II*** model sensitivity to *K_c_*. *K_c_* values of 0.1, 1, 10, and 100 were used, with V_tot_ *K_d_* = 1. The darkest lines refer to *K_c_* = 100. Black lines indicate doubly reduced species (V^0^), grey lines indicate unreduced species (V^2+^), yellow lines indicate singly reduced radical (V^+•^), and brown lines indicate singly reduced dimer ($V_{2}^{+•}$). d) ***Case II*** model sensitivity to V_tot_ *K_d_*. V_tot_ *K_d_* values of 0.1, 1, 10, and 100 were used, with *K_c_* = 1. The darkest lines refer to V_tot_ *K_d_* = 100. *x*_i_ refers to mole fraction. As before, black lines indicate doubly reduced species (V^0^), grey lines indicate unreduced species ($V^{2+}$), yellow lines indicate singly reduced radical (V^+•^), and brown lines indicate singly reduced dimer ($V_{2}^{+•}$).

In pyridinium electrolytes, the balance of species at a given SOC can be estimated by equilibrium modelling.^[^ ^5^, ^6^, ^7^, ^8^ ^]^ However, current modelling frameworks either only account for a single equilibrium process^[^ ^7^ ^]^ or apply simplifying assumptions intended to enable solutions for multiple equilibrium processes but only at specific SOCs (*e.g*., 0% SOC, 50% SOC, or 100% SOC),^[^ ^5^, ^8^ ^]^ not generally. In each case, there is insufficient information to capture the inherent complexity of pyridinium systems or to enable subsequent modelling of more complex phenomena including side reactions with oxygen. Thus, to arrive at a revised modelling framework capable of handling all processes of interest at all SOCs, two distinct cases were considered: i) major equilibrium processes (i.e., comproportionation and dimerization) occur simultaneously and on comparable timescales (***Case I***) ii) major equilibrium processes occur sequentially (i.e., one process is more fundamental and establishes dynamic equilibrium well before the other; ***Case II***) (**Figure** **1**a). We define the equilibria of interest for a generic viologen, V, that can accept two electrons (i.e., has the following oxidation states: V^2+^, V^+•^ and V^0^) as follows:

(1) $$V_{\left(\mathrm{aq}\right)}^{2+}+V_{\left(\mathrm{aq}\right)}^{0}⇌2V_{\left(\mathrm{aq}\right)}^{+•},K_{c}=\frac{{\left[V_{\left(\mathrm{aq}\right)}^{+•}\right]}^{2}}{\left[V_{\left(\mathrm{aq}\right)}^{2+}\right]\left[V_{\left(\mathrm{aq}\right)}^{0}\right]}$$

(2) $$2V_{\left(\mathrm{aq}\right)}^{+•}⇌{\left(V^{+•}\right)}_{2\left(\mathrm{aq}\right)},K_{d}=\frac{\left[{\left(V^{+•}\right)}_{2\left(\mathrm{aq}\right)}\right]}{{\left[V_{\left(\mathrm{aq}\right)}^{+•}\right]}^{2}}$$

In ***Case I***, assuming a two‐electron redox as is characteristic for pyridinium RFBs, and galvanostatic cycling conditions, equations for reduced species as a function of SOC take the form of fourth order polynomials (see derivations in Section S2, Supporting Information). Solutions to these yield concentration profiles that agree well with previously published experimental electron paramagnetic resonance spectroscopy (EPR) and ultraviolet‐visible spectroscopy (UV‐vis) based concentration profiles for methyl viologen (Figure 1a,b; Section S3, Supporting Information),^[^ ^4^, ^5^ ^]^ but that fail to account for more subtle features especially near the radical concentration maximum and at intermediate SOCs.

These results suggest that in the case of methyl viologen (MV) and possibly other pyridinium species, equilibrium processes may not occur on similar timescales, in line with prior experimental observations.^[^ ^9^, ^10^ ^]^ To account for this, a second approximate case, ***Case II***, was considered wherein comproportionation first occurs rapidly to yield an initial quantity of monoradical. This quantity of monoradical then much more slowly partitions into equilibrium quantities of monoradical and π‐dimer, respectively, such that conversion of π‐dimer back to monoradical is slow. Solutions to the resulting equations yield nested quadratics (Section S2, Supporting Information), which produce better agreement with experimental data both for methyl viologen (Figure 1a,b) and other more complex pyridinium species (Figure S1, Supporting Information), suggesting robustness of the underlying assumptions and confirming that the system is indeed under equilibrium conditions during cycling. Given that *K_d_* is calculated from both *K_c_* and the experimental radical concentration maximum, which can be measured either using *operando* spectroscopic methods (nuclear magnetic resonance spectroscopy (NMR),^[^ ^7^ ^]^ EPR,^[^ ^8^ ^]^ UV–vis,^[^ ^11^ ^]^ *etc*.), as well as by bulk magnetic susceptibility measurements,^[^ ^12^ ^]^ *K*_*d*_ values were calculated based on previously reported *K_c_* and maximum radical concentration data^[^ ^4^, ^5^ ^]^ using both ***Case I*** and ***Case II*** assumptions and compared (**Table** **1**). While agreement between *K*_*d*_ values was excellent at high *K_c_* values, larger deviations were observed at *K_c_* < 100, suggesting that choice of modelling approach can affect calculated values of *K*_*d*_. Nevertheless, once one set of assumptions is consistently used, agreement with experimental data remains robust.

**Table 1:** Comparison of *K*_d_ values calculated using *Case I* and *Case II* assumptions.

Compound	*K*_d_ [mM^−1^]		*K_c_*
*Case I*	*Case II*
**10**	0.28	0.28	3.2 × 10^5^
**11**	11	3.1	1.3 × 10^0^
**13**	76	32	2.9 × 10^0^
**17**	11	7.7	1.1 × 10^2^
**MV**	1.8	1.9	1.9 × 10^7^

## 3 Empirical and Theoretical Basis for Air Stability in Pyridinium Redox Flow Batteries

**Figure 2:**
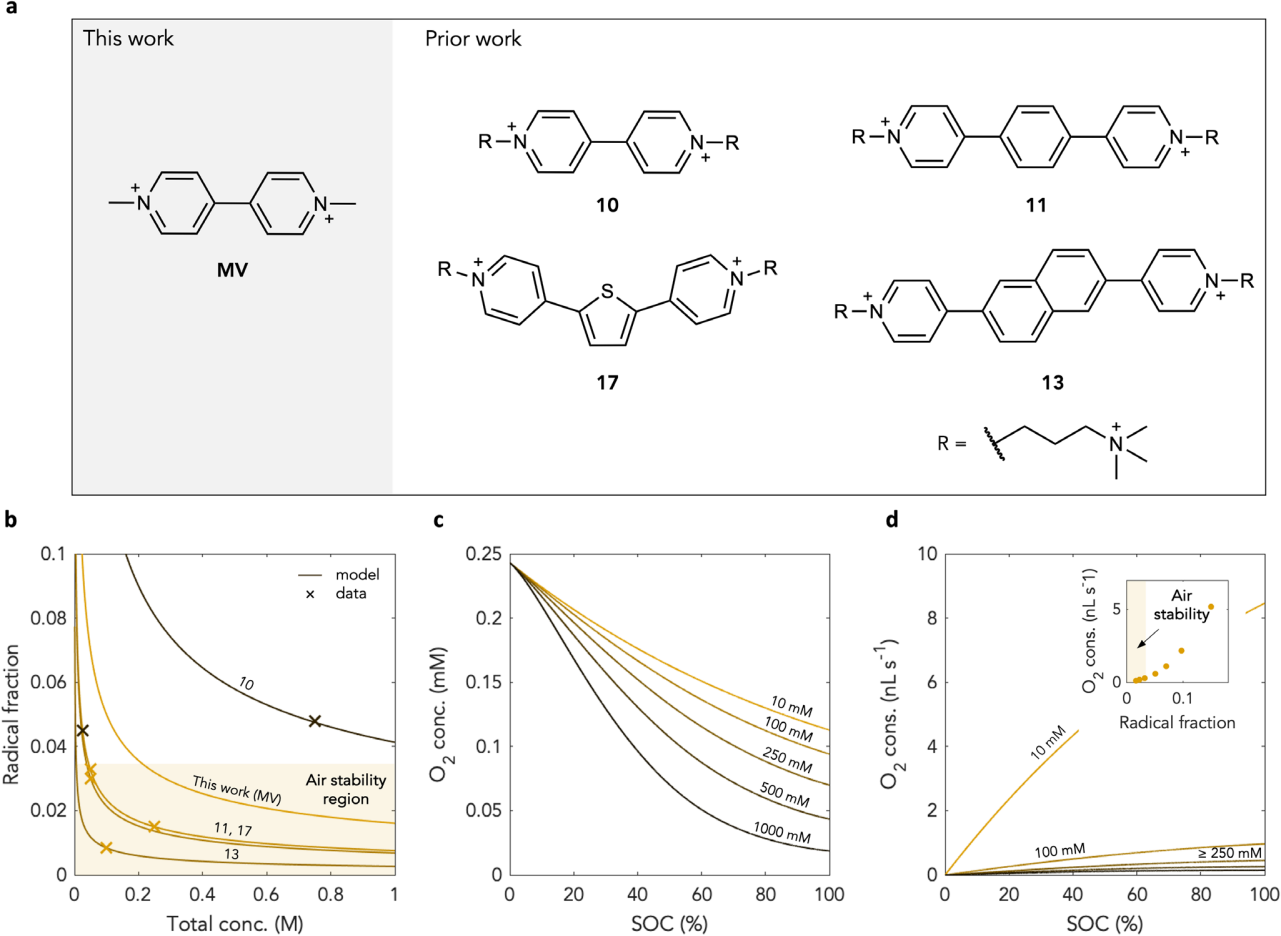
Radical concentration sensitivity and the role of low radical fractions in predicting air stability. a) Structures for representative pyridinium species for which air stability was previously tested.^[^ ^4^, ^5^, ^14^ ^]^ The compound labels correspond to those previously used.^[^ ^4^, ^5^ ^]^ b) Maximum radical fraction (***Case II*** assumptions) and the proposed onset of air stability on the basis of reported experimental data. Prediction for air stability of methyl viologen above a total system concentration of 200 mM. Crosses indicate experimental data. Lines indicate model predictions. Compounds considered include previously reported compounds **10** ^14^, **11** ^5^, **13** ^4^, and **17** ^5^. Black crosses indicate reported air instability. Yellow crosses indicate reported air stability. c) Model‐derived oxygen concentration as a function of SOC at 10 mM, 100 mM, 250 mM, 500 mM, and 1000 mM. d) Oxygen consumption per mole of active material as a function of SOC at 10 mM, 100 mM, 250 mM, 500 mM, and 1000 mM. Oxygen consumption per mole of active material as a function of maximum radical fraction at 10 mM, 25 mM, 50 mM, 100 mM, 250 mM, 500 mM, and 1000 mM (inset). At each concentration, oxygen consumption per mole of active material is calculated at 50% SOC (i.e., the SOC at which the radical concentration is highest). This oxygen consumption is then plotted against the maximum radical fraction at that concentration. Within the stability air stability threshold, there is almost no change in molar oxygen consumption with total system concentration, and oxygen consumption per mole of active material is approximately linearly related to maximum radical fraction.

With a modelling framework developed, an investigation into the factors that influence air stability was next conducted. Recent work has suggested that a higher concentration of unpaired radicals loosely correlates with a higher degree of capacity fade due to parasitic processes including reaction with oxygen.^[^ ^4^, ^5^ ^]^ In order to further probe this, analysis of maximum radical fraction was next conducted as the normalised (and dimensionless) analogue of maximum radical concentration. Plotting experimentally determined maximum radical fraction as a function of total system concentration for four representative pyridinium species (**Figure** **2**a), for which air stability was previously experimentally tested,^[^ ^4^, ^5^, ^13^ ^]^ revealed an apparent threshold for stability (Figure 2b). This occurred at a maximum radical fraction of ≈0.033 (Figure 2b) and for compounds that possessed both homo‐ and heterocyclic cores, varying degrees of conjugation, and both classical and “extended” cores, suggesting generality (Figure 2a).

To examine the role of oxygen in the system more fully, the modelling framework developed above was then expanded to take into account the presence of dissolved oxygen from which a more complete understanding of the system could be gained. It should be noted here that while reduction of molecular dioxygen leads to complex products including reactive oxygen species (ROS), which can aslo contribute to capacity fade,^[^ ^5^, ^13^ ^]^ for simplicity only reactions with dissolved O_2_ are considered – all downstream products are assumed to be deleterious to the system. Assuming further that at sufficiently high concentrations, the presence of dissolved oxygen does not substantially affect concentration profiles for V^2+^, $V^{+•}$, V^0^, and $V_{2}^{+•}$, as confirmed by *operando* NMR‐based Evans method experiments at 100 mM (Figure S2; Section S3, Supporting Information), a mass balance on dissolved oxygen yields the following simplified differential equation:

(3) $$\begin{array}{ccc} \frac{d\left[O_{2}\right]}{dt} & + & \left[O_{2}\right]\;\left(\frac{Ak_{L}+S_{A}k_{\mathrm{dir},O2}+Vk_{O2,\;V+}\left[V^{+•}\right]+Vk_{O2,\;V0}\left[V^{0}\right]}{V}\right)\\ & & =\frac{Ak_{L}C_{O2,\mathrm{int}}}{V} \end{array}$$

where [O_2_] is the bulk concentration of dissolved oxygen; *C*_O2,int_ is the concentration of dissolved oxygen at the air‐liquid interface due to Henry's law; *A* is the interfacial area exposed to air; *S*_A_ is the interfacial area exposed to the electrode; *k*_dir,O2_ is the rate constant for reduction of dissolved oxygen at an electrode; *k*_O2,V+_ and *k*_O2,V0_ are the second order rate constants for reduction of dissolved oxygen by $V^{+•}$ and V^0^, respectively; and *k*_L_ is the mass transfer coefficient for transport of oxygen across the air‐liquid interface. For simplicity, activity coefficients are assumed to be close to unity (a reasonable assumption under the low to intermediate concentrations used for experimental validation of the parent ***Case I*** & ***II*** models, but one expected to break down under high concentrations).

Solving the above equation for [O_2_], [O_2_] was found to decrease both with SOC and total pyridinium concentration (Figure 2c) as expected. However, by analogy to the case of radicals, the capacity fade rate is believed to correlate with the fraction of active material participating in parasitic processes – in this case, oxygen reduction. Calculating the equilibrium rate of oxygen transported into the system and normalising by total pyridinium concentration yielded a trend in line with radical‐based air stability predictions (Figure 2d). At a concentration of 10 mM, oxygen consuption per mol of active material was found to be substantial (in line with a maximum radical fraction of 0.14) indicating little to no air stability. At a concentration of 100 mM, corresponding to a maximum radical fraction of 0.050, i.e., just outside the air stability threshold (Figure 2b), oxygen consuption per mol of active material was found to be much lower, but still notable. At total system concentrations of 250 mM and above, i.e., below a radical fraction of 0.033 (the air stability threshold), oxygen consumption per mol of active material became almost negligible and, moreover, insensitive to SOC in agreement with empirical trends. Indeed, plotting oxygen consumption per mol of active material directly against maximum radical fraction (Figure 2d, inset) revealed a monotonic correlation indicating minimal, approximately linear increases in molar oxygen consuption within the air stability region, but rapid increases thereafter. Thus, the observed empirical trend can be directly rationalised on the basis of predicted oxygen consumption rates, and therefore has a theoretical basis.

## 4 Validation and Compatibility with Long Duration Energy Storage Scaling Requirements

**Figure 3:**
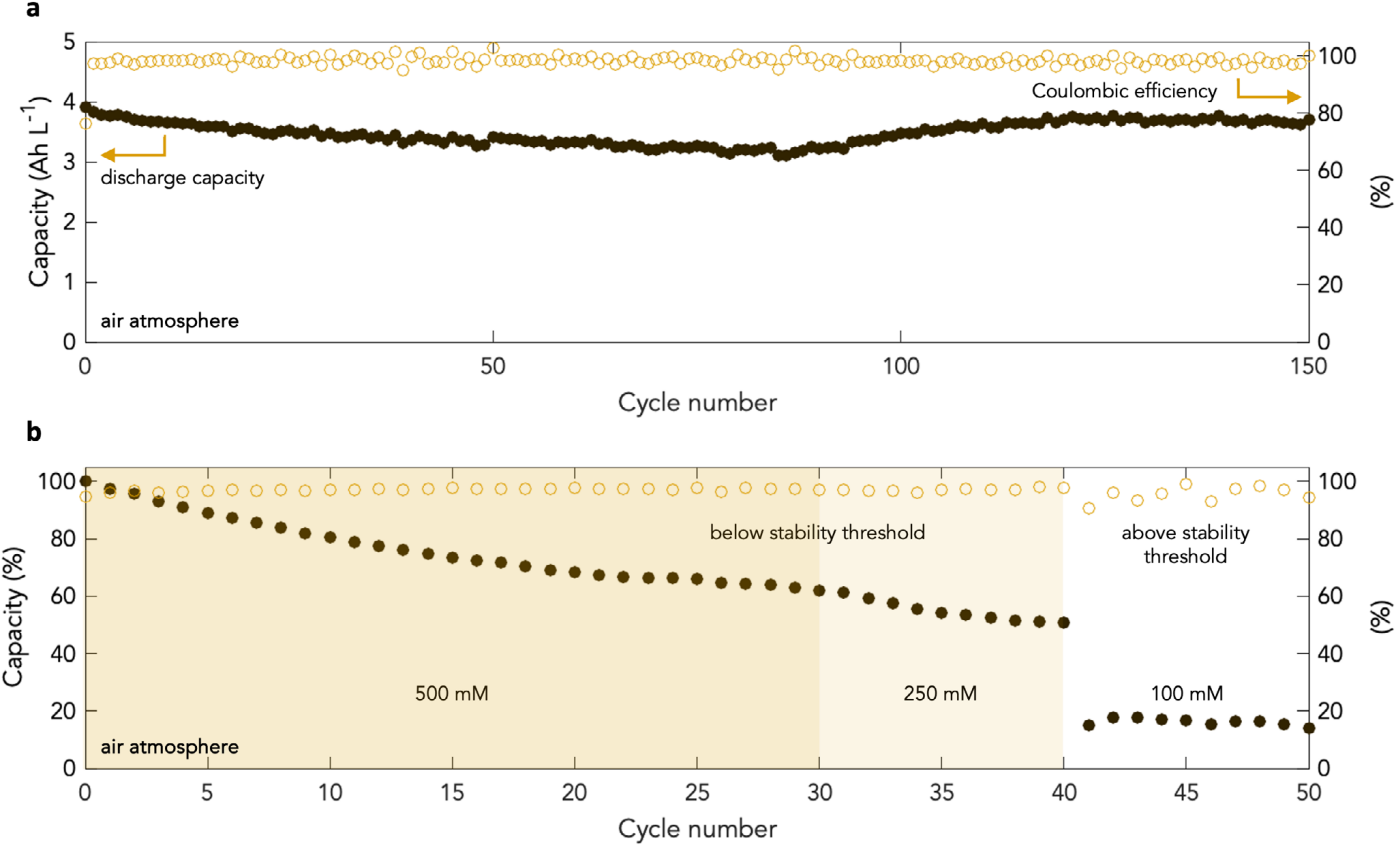
Stability of methyl viologen in air. a) Discharge capacity and Coulombic efficiency of a 250 mM MV and 250 mM 4‐hydroxy‐TEMPO in 1 M NaCl full cell cycled 150 times in air at a current density of 40 mA cm^− 2^. Cut‐off voltages of 0.5 and 1.55 V were used. b) Discharge capacity and Coulombic efficiency of a 500 mM MV and 250 mM 4‐hydroxy‐TEMPO in 1 M NaCl full cell cycled 30 times in air at a current density of 40 mA cm^−2^. The anolyte was then diluted to 250 mM and cycled a further 10 times. Finally, the anolyte was diluted to 100 mM and cycled a further 10 times. Cut‐off voltages of 0.5 and 1.55 V were used as before.

To test and validate the predictive power of such a trend, methyl viologen, the cheapest and most well‐studied pyridinium electrolyte to date, should exhibit air stability above a total system concentration of 200 mM (Figure 2b). Thus, full cells consisting of a MV anolyte and a 4‐OH‐TEMPO catholyte were assembled and tested at various system concentrations. At a system concentration of 250 mM, methyl viologen is predicted to be air stable. At a current of 40 mA cm^−2^, methyl viologen was cycled 150 times in air under one‐electron operation (**Figure** **3**a). Over the 150 cycles run, capacity retention was found to be robust with the system exhibiting 94.9% capacity retention (0.034% fade per cycle; 2.3% fade per day) and a Coulombic efficiency of 98.3 ± 1.3%. These values are in line with those obtained for methyl viologen under similar conditions but under a strict air‐free environment,^[^ ^14^ ^]^ confirming robust air stability. With air stability demonstrated, cell failure outside of the air stability window was next tested. For these experiments, at 40 mA cm^−2^, a full cell consisting of a methyl viologen anloyte and a 4‐OH‐TEMPO catholyte was cycled until stability was reached, starting from a total system concentration of 500 mM (Figure 3b). At 500 mM, as a result of the low current used, performance initially decayed then stabilised at around the 30th cycle. It should be noted that this decay is consistent with that previously observed for methyl viologen at 500 mM under a strict air‐free environment when paired with a 4‐OH‐TEMPO catholyte.^[^ ^14^ ^]^ At this stage, oxygenated (i.e., undegassed) water was added to the anloyte to dilute it to 250 mM ahead of further cycling. After another slight performance decay, the system stabilised within 10 cycles to achieve capacities in line with those obtained in the previous extended run. Remarkably, however, when the system was diluted to 100 mM, a dramatic step decline in performance was observed confirming a lack of air stability below 250 mM for MV in line with empirical and theoretical predictions. Thus, the thresholds presented above can be used to successfully predict air stability and air‐induced failure in pyridinium electrolytes.

**Figure 4:**
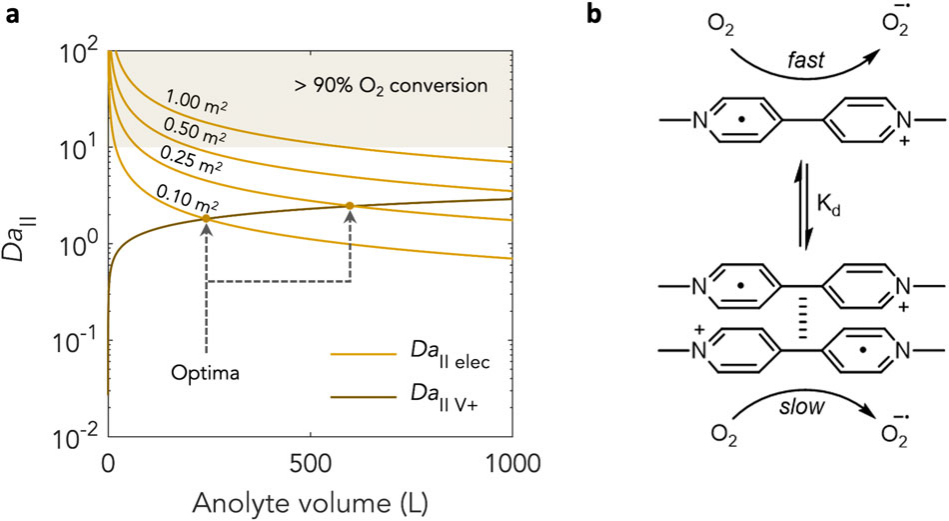
System requirements for low conversion of oxygen at scale. a) Second Damköhler number sensitivity to electrolyte volume at different electrode areas. The dashed arow indicates optimal *V* to *A* scaling. b) Scheme showing previously underappreciated association‐mediated kinetic arguments for suppression of reactivity with oxygen.

With robust methods developed for predicting and rationalising air stability, compatibility with systems level requirements for long duration energy storage (LDES) was next sought (**Figure** **4**). LDES applications require high discharge durations at rated power, that is, discharge durations typically in excess of 10 h.^[^ ^15^ ^]^ For a redox flow battery system architecture wherein electrolyte tanks can be scaled independently of cell stacks, long durations can be achieved at high energy to power ratios.^[^ ^16^ ^]^ Given that energy scales with electrolyte volume and power scales with electrode area,^[^ ^16^ ^]^ low oxygen conversions at high ratios of volume to electrode area are needed to demonstrate compatibility for LDES applications. Starting from equation 3 above, dimensional analysis yields four dimensionless time constants that can be further consolidated into three dimenionless groups:

(4) $$Da_{\mathrm{II}\;\mathrm{elec}}=\frac{S_{A}k_{\mathrm{dir},O2}}{Ak_{L}}Da_{\mathrm{II}\;V+}=\frac{Vk_{O2,\;V+}\left[V^{+•}\right]}{Ak_{L}}Da_{\mathrm{II}\;V0}=\frac{Vk_{O2,\;V0}\left[V^{0}\right]}{Ak_{L}}$$

where *Da*_II_ is the second Damköhler number^[^ ^17^, ^18^ ^]^ – a dimensionless group of constants that describes the rate of reaction relative to the rate of mass transfer. Damköhler numbers are widely used in chemical reaction engineering to gain an initial estimate of the degree of conversion that can be achieved for a compound of interest; the higher the value of *Da*_II_, the higher the degree of conversion. As a rule of thumb, a *Da*_II_ of 10 roughly corresponds to 90% conversion.^[^ ^18^ ^]^ Within the context of the current system, *Da*_II_ describes the rate of oxygen consumption due to electrode reduction (*Da*_II elec_), reaction with V^+•^ (*Da*_II V+_), or reaction with V^0^ (*Da*_II V0_) to the rate of oxygen transported into the system. It should be noted that *Da*_II V+_ is directly proportional to radical fraction, and thus, can be tied to the air stability threshold.

Inspection of *Da*_II_ reveals that *Da*_II elec_ scales with electrode area (*A*), whereas *Da*_II V+_ and *Da*_II V0_ scale with electrolyte volume (*V*). Thus, there are independent oxygen conversion scalings for different ratios of energy to rated power. To gain insight into the nature of these scalings, *Da*_II elec_ and *Da*_II V+_ were next evaluated as a function of electrolyte volume for a *K_d_* of 100 mM^−1^ and a *K_c_* of 1 for different electrode areas. A system concentration of 1.5 M, a fixed standard tank aspect ratio of 1, and rate constants similar to those of methyl viologen in the first instance were assumed (Figure 4a). Under these conditions, *Da*_II V+_ was found to increase with volume.

This increase was sensitive to *K_d_*, with higher *K_d_* values favouring lower *Da*_II_ values, as expected (Figure S3, Supporting Information). By contrast, *Da*_II V+_ was found to be almost completely insensitive to *K_c_* even when *K_c_* was varied over ten orders of magnitude (Figure S3, Supporting Information). *Da*_II V+_ was also found to be acutely sensitive to *k*_O2,V+_, where a decrease from 2.2 × 10^−6^ to 2.2 × 10^−7^ mM^−1^ s^−1^ was enough to decrease *Da*_II_ by an order of magnitude (Figure S4, Supporting Information). Given that rates of electron transfer from unpaired radicals to oxygen are expected to be higher than for paired or encapsulated^[^ ^19^, ^20^ ^]^ radicals to oxygen, at sufficiently high *K_d_* values or under regimes of sufficently fast association, it may be possible to effectively lower the observed value of *k*_O2,V+_ in solution. Thus, a high system propensity for association can have added kinetic benefits in suppressing side processes with air (Figure 4b). As association can be expediently tuned through choice of solubilising R‐groups, covalent tethering and through electrolyte formulation,^[^ ^3^, ^20^, ^21^, ^22^ ^]^ such strategies hold promise for robust design of air stable systems at full scale.

While *Da*_II V+_ increased with volume, *Da*_II elec_ decreased as a result of the increased interfacial area exposed to air. This decrease became more pronounced as electrode area was systematically decreased to 0.1 m^2^ (Figure 4a). Given this general decrease, coupled with the general increase of *Da*_II V+_ under the same scaling, a design paradigm emerges where the optimal volume to electrode area scaling can be found from the intersection of the *Da*_II elec_ and *Da*_II V+_ curves as indicated in Figure 4a – that is, the optimum respresents the point at which the Damköhler number (effectively, dimensionless O_2_ conversion) is at its global minimum. Given further that the identified optimum is consistent with an LDES scaling (i.e., as characterised by a high volume to electrode area ratio), and that *Da*_II V+_ increases only very gently with volume above the optimum scaling, high discharge durations can be accessed while still operating safely within air stability thresholds, confirming consistency with LDES scaling requirments.

## 5 Conclusion

In the present work, we developed a robust modelling framework that not only provided new insights into the factors that govern species evolution with SOC but also enabled the development of simple but powerful methods for predicting air stability in pyridinium RFBs. These methods were used to correctly predict the air stability of methyl viologen – the lowest cost and most well studied pyridinium species for RFB applications to date. They were also used to correctly predict the onset of air‐induced failure within methyl viologen‐based systems. By derivation of the second Damköhler number, a dimensionless parameter describing the rate of reaction relative to the rate of mass transfer, factors that promote air stability were shown to be consistent with scaling requirements needed for application in LDES. Given that many classes of organic electrolyte undergo comproportionation and dimerization equilibria including quinones and aza‐aromatics,^[^ ^7^, ^8^, ^23^, ^24^, ^25^ ^]^ it is anticipated that the theoretical framework and results presented herein will have broad applicability toward the development of air stable organic electrolytes moving forward. It is also expected that this work will serve as a starting point for future systems level engineering of RFBs for air stable LDES.

## Conflict of Interest

The authors declare no conflict of interest.

## Supporting information



Supporting Information

## Data Availability

The data that support the findings of this study are available from the corresponding author upon reasonable request.

## References

[1] C. L. Bird , A. T. Kuhn , Chem. Soc. Rev. 1981, 10, 49.

[2] K. Sokolowski , J. Huang , T. Földes , J. A. McCune , D. D. Xu , B. de Nijs , R. Chikkaraddy , S. M. Collins , E. Rosta , J. J. Baumberg , O. A. Scherman , Nat. Nanotechnol. 2021, 16, 1121. 34475556 10.1038/s41565-021-00949-6

[3] O. Nolte , R. Geitner , I. A. Volodin , P. Rohland , M. D. Hager , U. S. Schubert , Adv. Sci. 2022, 9, 2200535. 10.1002/advs.202200535 PMC9189600 35481674

[4] M. E. Carrington , Supramolecular effects in pyridinium compounds and their application towards practical redox flow battery systems 2024, University of Cambridge.

[5] M. E. Carrington , K. Sokolowski , E. Jónsson , E. W. Zhao , A. M. Graf , I. Temprano , J. A. McCune , C. P. Grey , O. A. Scherman , Nature 2023, 623, 949. 38030777 10.1038/s41586-023-06664-7 PMC10686829

[6] L. Michaelis , G. F. Boeker , R. K. Reber , J. Am. Chem. Soc. 1938, 60, 202.

[7] E. W. Zhao , T. Liu , E. Jonsson , J. Lee , I. Temprano , R. B. Jethwa , A. Wang , H. Smith , J. Carretero‐Gonzalez , Q. Song , C. P. Grey , Nature 2020, 579, 224. 32123353 10.1038/s41586-020-2081-7

[8] E. W. Zhao , E. Jónsson , R. B. Jethwa , D. Hey , D. Lyu , A. Brookfield , P. A. A. Klusener , D. Collison , C. P. Grey , J. Am. Chem. Soc. 2021, 143, 1885. 33475344 10.1021/jacs.0c10650 PMC7877726

[9] C. W. Lee , J. C. Eklund , R. Dryfe , R. G. Compton , Bull. Korean Chem. Soc. 1996, 17, 162.

[10] P. M. S. Monk , N. M. Hodgkinson , S. A. Ramzan , Dyes Pigm. 1999, 43, 207.

[11] L. Tong , Q. Chen , A. A. Wong , R. Gómez‐Bombarelli , A. Aspuru‐Guzik , R. G. Gordon , M. J. Aziz , Phys. Chem. Chem. Phys. 2017, 19, 31684. 29165500 10.1039/c7cp05881k

[12] E. W. Zhao , E. J. K. Shellard , P. A. A. Klusener , C. P. Grey , Chem. Commun. 2022, 58, 1342. 10.1039/d1cc01895g 34986212

[13] E. S. Beh , D. De Porcellinis , R. L. Gracia , K. T. Xia , R. G. Gordon , M. J. Aziz , ACS Energy Lett. 2017, 2, 639.

[14] T. Liu , X. Wei , Z. Nie , V. Sprenkle , W. Wang , Adv. Energy Mater. 2016, 6, 1501449.

[15] P. Albertus , J. S. Manser , S. Litzelman , Joule 2020, 4, 21.

[16] R. M. Darling , K. G. Gallagher , J. A. Kowalski , S. Ha , F. R. Brushett , Energy Environ. Sci. 2014, 7, 3459.

[17] G. Damköhler , Z. Elektrochem. Angew. Phys. Chem. 1936, 42, 846.

[18] H. S. Fogler , Elements of Chemical Reaction Engineering (4th ed.). Prentice Hall, Upper Saddle River, NJ 2006.

[19] J. C. Barnes , A. C. Fahrenbach , D. Cao , S. M. Dyar , M. Frasconi , M. A. Giesener , D. Benítez , E. Tkatchouk , O. Chernyashevskyy , W. H. Shin , H. Li , S. Sampath , C. L. Stern , A. A. Sarjeant , K. J. Hartlieb , Z. Liu , R. Carmieli , Y. Y. Botros , J. W. Choi , A. M. Z. Slawin , J. B. Ketterson , M. R. Wasielewski , W. A. Goddard , J. F. Stoddart , Science 2013, 339, 429. 23349286 10.1126/science.1228429

[20] J. Sun , Z. Liu , W.‐G. Liu , Y. Wu , Y. Wang , J. C. Barnes , K. R. Hermann , W. A. Goddard , M. R. Wasielewski , J. F. Stoddart , J. Am. Chem. Soc. 2017, 139, 12704. 28806074 10.1021/jacs.7b06857

[21] Z. Xiang , W. Li , K. Wan , Z. Fu , Z. Liang , Angew. Chem., Int. Ed. 2023, 135, 202214601. 10.1002/anie.202214601 36383209

[22] M. R. Geraskina , A. S. Dutton , M. J. Juetten , S. A. Wood , A. H. Winter , Angew. Chem., Int. Ed. 2017, 129, 9563. 10.1002/anie.201704959 28635187

[23] J.‐M. Lü , S. V. Rosokha , J. K. Kochi , J. Am. Chem. Soc. 2003, 125, 12161. 14519002 10.1021/ja0364928

[24] M. J. Gibian , R. C. Corley , Chem. Rev. 1973, 73, 441.

[25] D. G. Kwabi , Y. Ji , M. J. Aziz , Chem. Rev. 2020, 120, 6467. 32053366 10.1021/acs.chemrev.9b00599

