# Supplementary material for: Design Principles for Air Tolerance in Pyridinium‐Based Flow Batteries
Source: Adv Mater. 2025 Nov 5;38(5):e08875. doi: 10.1002/adma.202508875 (PMC12822527; doi:10.1002/adma.202508875)
Supplement: Supplementary file 1 — Supporting Information [file ADMA-38-e08875-s001.docx]

Supporting Information

Title: Design principles for air tolerance in pyridinium-based flow batteries

*Mark E. Carrington, Erlendur Jónsson, Clare P. Grey ^*^*

**Table of contents**

**S1.** Materials and methods

**S2.** Thermodynamic and kinetic modelling

**S3.** EPR and UV-Vis data

**S4.** References

**S1. Materials and methods**

*Materials*

Methyl viologen chloride (98%) and sodium chloride (99%) were purchased from Fisher Scientific. **4-hydroxy-2,2,6,6-tetramethylpiperidine-1-oxyl (97%) was** purchased from Sigma-Aldrich. Milli-Q water was used for preparation of all non-deuterated aqueous solutions. Materials were used as obtained without further purification.

*Cell assembly*

The hardware of the flow battery was purchased from Scribner Associates. Ultrahigh-purity sealed graphite flow plates with serpentine flow patterns were used for both electrodes. Each electrode comprised 4.6 mm carbon felt (SGL) with a 5 cm^2^ active area. Selemion AMVN was used as the anion exchange membrane. PTFE frames with a thickness of 3 mm were used to position the electrodes with Viton gaskets 0.7 mm in thickness on each side of the frames. The current collectors were gold-plated copper plates. Anodized aluminium end plates with reactant input/output ports were used.

*Operando NMR experimental protocol*

The setup was as previously reported.^1–3^ Briefly, the setup consisted of a flow battery, two peristaltic pumps, an electrochemical cycler (SP-150, BioLogic SAS), and a NMR (300 MHz, Bruker Avance IIIHD) spectrometer. The battery was positioned outside the 5 G line of the NMR magnet. The electrolyte was pumped through the flow battery, then flowed through the NMR magnet and finally back to the electrolyte reservoir. In the anolyte reservoir, the ﬂow cell employed 30 mL of a 0.10 M methyl viologen in 1.0 M NaCl aqueous solution. In the catholyte reservoir, the ﬂow cell employed 50 mL of a 0.2 M 4-hydroxy-TEMPO in 1.0 M NaCl aqueous solution. Both reservoirs were purged with N_2_, degassed for 1 h and then kept under active N_2_ flow during cycling. The ﬂow cell was galvanostatically charged and discharged at room temperature on a portable electrochemical cycler at current of 100 mA (20 mA cm^-2^) between voltages of 0.5 V & 2.05 V. A high potential hold of 1 h was implemented at 2.05 V and a low potential hold of 2h was implemented at 0.5 V. After two complete charge discharge cycles, the nitrogen ports were disconnected, leaving the cycling cell under static exposure to air. Pseudo-2D NMR experiments were performed on the flowing electrolyte solution by direct excitation with a 90° radiofrequency pulse. Each NMR spectrum was acquired by collecting eight free induction decays (FIDs) with a recycle delay of 15 s. The pulse width for a 90° pulse was 27 μs at 30 W. All spectra were referenced to the water chemical shift at 4.79 ppm. Quantification of radical concentration was based on state of charge dependent changes in bulk magnetisation as quantified by changes in the water chemical shift, as previously described.^1^ A constant of proportionality between chemical shift and radical concentration of 183.2 mol m^-3^ was used. Electrochemical data were processed using EC-lab v.11.50 (BioLogic).

*Extended cycling under air*

For extended galvanostatic cycling studies, in the anolyte reservoir, the ﬂow cell employed either 10 mL of a 0.25 M methyl viologen in 1.0 M NaCl aqueous solution or 10 mL of a 0.50 M methyl viologen in 1.0 M NaCl aqueous solution. In the catholyte reservoir, the ﬂow cell employed either 30 mL or 60 mL, respectively, of a 0.25 M 4-hydroxy-TEMPO in 1.0 M NaCl aqueous solution. Cut-off voltages of 0.5 V and 1.55 V were used. The ﬂow cell was galvanostatically cycled as before using a portable electrochemical cycler (SP-150, BioLogic SAS). During cycling, each electrolyte reservoir was kept open to atmosphere by way of a standard laboratory needle (18 gauge) to ensure continuous replenishment of the air atmosphere in the headspace above the electrolyte, but limited solvent evaporation during cycling. All cells were assembled and run under ambient conditions in the presence of atmospheric air. Electrochemical data were processed using EC-lab v.11.50 (BioLogic).

*Case II model fits to experimental EPR data*

**Figure S1 | *Case II* fits for other pyridinium species.** Radical concentration predictions for **(a)** **10^+•^**, **(b)** **17^+•^**, and **(c)** **11^+•^** as a function of SOC compared with prior experimental data derived from EPR spectroscopy.^3,4^

*Operando NMR results confirming radical profile insensitivity to atmosphere*

**Figure S2 | Radical concentration data for MV from NMR at 100 mM both under N_2_ and air atmospheres.** The radical concentration was extracted directly from state of charge dependent changes in bulk magnetisation as quantified by changes in the water chemical shift, as previously described.^1^ The ﬂow cell employed 30 mL of a 0.10 M methyl viologen in 1.0 M NaCl aqueous solution as anolyte. As catholyte, the ﬂow cell employed 50 mL of a 0.2 M 4-hydroxy-TEMPO in 1.0 M NaCl aqueous solution. Both reservoirs were purged with N_2_, degassed for 1 h and then kept under active N_2_ flow during cycling. The ﬂow cell was galvanostatically charged and discharged at room temperature at current of 100 mA (20 mA cm^-2^) between voltages of 0.5 V & 2.05 V. A high potential hold of 1 h was implemented at 2.05 V and a low potential hold of 2h was implemented at 0.5 V. After two complete charge discharge cycles, the nitrogen ports were disconnected, leaving the cycling cell under static exposure to air.

**S2. Thermodynamic and kinetic modelling**

***Case I:* equilibrium processes all occur simultaneously**

Assuming a fully soluble two-electron system under equilibrium subject to comproportionation and dimerisation, electron accounting gives:

$$e_{tot}=n_{V+\cdot}+2n_{Vdim}+ 2 n_{V0} (S1)$$

where *e*_tot_ is moles of electrons, *n*_V+•_ is moles of singly reduced species, *n*_Vdim_ is moles of dimer, and *n*_V0_ is moles of doubly reduced species. State of charge (SOC) is then:

$$SOC=\frac{e_{tot}}{2 n_{Vtot}} (S2)$$

where:

$$n_{Vtot}=n_{V2+}+ n_{V+\cdot}+ n_{Vdim}+2 n_{V0} (S3)$$

and *n*_Vtot_ is total starting moles of the redox active material. To determine moles of radical (which can be experimentally benchmarked using a variety of methods) as a function of SOC, rearranging the equations above gives:

$$n_{V+\cdot}=2 SOC n_{Vtot}- 2 n_{Vdim}-2 n_{V0} (S4)$$

Now, comproportionation and dimerisation equilibria require:

$$V_{(aq)}^{2+}+V_{(aq)}^{0}\rightleftharpoons2V_{(aq)}^{+\bullet}, K_{c}=\frac{\left[ V_{(aq)}^{+\bullet} \right]^{2}}{\left[ V_{(aq)}^{2+} \right]\left[ V_{(aq)}^{0} \right]} (S5)$$

$$2V_{(aq)}^{+\bullet}\rightleftharpoons\left( V^{+\bullet} \right)_{2 (aq)},K_{d}=\frac{\left[ \left( V^{+\bullet} \right)_{2 (aq)} \right]}{\left[ V_{(aq)}^{+\bullet} \right]^{2}} (S6)$$

Expressing (S5) & (S6) in terms of moles gives:

$$n_{V+\cdot}^{2}=K_{c} n_{V2+}n_{V0} (S7)$$

$$n_{Vdim}=K_{d}\frac{n_{V+\cdot}^{2}}{V} (S8)$$

where *V* is volume. Substituting into (S3), then gives:

$$-n_{V0}^{2}+n_{V0}(n_{Vtot}-n_{V+\cdot}-2 n_{Vdim})-\frac{n_{V+\cdot}^{2}}{K_{c}}=0 (S9)$$

which is a quadratic equation. Solving this and substituting into (S4) gives:

$$0=2 SOC n_{Vtot}-n_{Vtot}\pm\sqrt{{({n_{Vtot}-n}_{V+\cdot}-2 n_{Vdim})}^{2}-4\frac{n_{V+\cdot}^{2}}{K_{c}}} (S10)$$

Rearranging, squaring both sides and substituting in (S8) gives the quartic:

$$0=4\frac{K_{d}^{2}}{V^{2}}n_{V+\cdot}^{4}+4\frac{K_{d}}{V}n_{V+\cdot}^{3}-4\left( \frac{{n_{Vtot}K}_{d}}{V}+\frac{1}{K_{c}}-\frac{1}{4} \right)n_{V+\cdot}^{2}-{2n_{Vtot}n_{V+\cdot}+n}_{Vtot}^{2}-n_{Vtot}^{2}{(1-2 SOC)}^{2}$$

This is then solved numerically. By inspection, if *K_d_* goes to 0 or if the system becomes very dilute, the equation above simplifies in a way that removes dimer components as would be expected. More specifically, if *K_d_* becomes 0, the equation above simplifies to:

$$0=-4\left( \frac{1}{K_{c}}-\frac{1}{4} \right)n_{V+\cdot}^{2}-{2n_{Vtot}n_{V+\cdot}+n}_{Vtot}^{2}-n_{Vtot}^{2}\left( 1-2 SOC \right)^{2} (S11)$$

which is a quadratic, identical to that previously derived considering only comproportionation, not dimerisation.^1^

***Case II:* comproportionation occurs first and subsequent dimerisation is relatively slow**

Starting from:

$$0=-4\left( \frac{1}{K_{c}}-\frac{1}{4} \right)n_{V+\cdot}^{2}-{2n_{Vtot}n_{V+\cdot}+n}_{Vtot}^{2}-n_{Vtot}^{2}{(1-2 SOC)}^{2} (S11)$$

which is identical to that previously reported^1^ for comproportionation,

$$n_{V+\cdot comp}=n_{V+\cdot ss}+{2 n}_{Vdimss} (S12)$$

and:

$$n_{Vdimss}=K_{d}^{'}\frac{n_{V+\cdot ss}^{2}}{V} (S13)$$

where *n*_V+•comp_ is identical to *n*_V+•_ assuming only comproportionation occurs, *n*_V+•ss_ is the steady state number of moles of radical after dimerisation, *n*_Vdimss_ is the steady state number of moles of dimer after dimerisation, and *K_d_’* is the dimerisation constant under ***Case II*** assumptions. Substituting (S13) into (S12) and rearranging gives:

$$0=2 K_{d}^{'}\frac{n_{V+\cdot ss}^{2}}{V}+n_{V+\cdot ss}-n_{V+\cdot comp} (S14)$$

Solving for steady state radical concentration then gives:

$$n_{V+\cdot ss}=\frac{-1\pm\sqrt{1+8\frac{K_{d}^{'}}{V}n_{V+\cdot comp}}}{4\frac{K_{d}^{'}}{V}} (S15)$$

Dimer concentration was then obtained by substituting the equation above back into equation (S12). In addition to the predictions shown in Fig.1, the equation above for moles of radical was also used to quantify maximum radical fraction values shown in Fig. 2a, using *K*_d_ values from Table 1. Using ***Case II*** assumptions, *n*_V+•comp_ at the maximum radical fraction is:

$$n_{V+\cdot comp}=\frac{n_{Vtot}}{\frac{2}{\sqrt{K_{c}}}+1} (S16)$$

**Reaction with oxygen**

Assuming that the anolyte system can be modelled as a tank with an air interface of area *A* and an electrode interface with area *S_A_*, the rate of O_2_ accumulation in the system is given by:

$$rate of accumulation of O_{2} =rate of transport accross the air/liquid interface-rate of direct consumption at the electrode surface-rate of consumption from bulk reaction with V^{+\cdot}-rate of consumption from bulk reaction with V^{0}$$

Thus:

$$\frac{V d[O_{2}]}{dt}=Ak_{L}(C_{O2,int}-[O_{2}])-S_{A}k_{dir,O2}[O_{2}]-Vk_{O2, V+}\left[ V^{+\bullet} \right][O_{2}]-Vk_{O2, V0}\left[ V^{0} \right][O_{2}] (S17)$$

where *k* corresponds to the relevant rate constant and *C*_O2,int_ is the concentration of dissolved oxygen at the air-liquid interface due to Henry’s law. If desired, the rate of direct O_2_ consumption at the electrode surface and the expression for the rate of transport across the air/liquid interface can be further elaborated to consider specific transport components. However, for simplicity, and to obviate the need for additional data on boundary layers, which may be difficult to obtain and dependent on other parameters (*e.g.*, flow rate), equation (S17) was used to provide initial system insights to a first approximation. Rearranging (S17) gives:

$$\frac{d[O_{2}]}{dt}+\left[ O_{2} \right]\left( \frac{Ak_{L}+S_{A}k_{dir,O2}+Vk_{O2, V+}\left[ V^{+\bullet} \right]+Vk_{O2, V0}\left[ V^{0} \right]}{V} \right)= \frac{Ak_{L}C_{O2,int}}{V} (S18)$$

Under galvanostatic cycling conditions, time can be converted to SOC according to:

$$SOC=\frac{It}{C_{bat}} (S19)$$

where I is current and *C*_bat_ is battery capacity. Substituting (S19) into (S18) and solving the differential equation using either ***Case I*** or ***Case II*** values for V^+•^ and V^0^ (assuming the equilibrium values for V^+•^ and V^0^ under inert conditions are similar to the equilibrium values for V^+•^ and V^0^ under air; see Fig. S1) allows evaluation of [O_2_] as a function of SOC (Fig. 2b). Values of constants used for quantification of [O_2_] as a function of SOC include:

**Table S1 | Values of constants used in kinetic model.**

| **Parameter** | **Value** | **Notes** |
| --- | --- | --- |
| *A* | 5 cm^2^ | Values representative of lab-scale flow cells |
| *S_A_* | 5 cm^2^ | Values representative of lab-scale flow cells |
| *V* | 30 mL | Values representative of lab-scale flow cells |
| *C*_O2,int_ | 0.24 mM | Henry’s law partitioning of O_2_ in pure water |
| *k*_O2,V+_ | 2.2 × 10^-6^ mM^-1^ s^-1^ | From prior literature^5^ |
| *k*_O2,V0_ | 2.2 × 10^-7^ mM^-1^ s^-1^ | As for methyl viologen, V^0^ adsorbs strongly onto the carbon electrode preventing direct quantification, *k*_O2,V0_ was assumed to be at least an order of magnitude lower than *k*_O2,V+•_ in bulk solution. |
| *k*_dir,O2_ | 8.4 × 10^-4^ cm s^-1^ | From prior literature^6^ |
| *k*_L_ | 1.3 × 10^-4^ cm s^-1^ | From prior literature^7^ |

From these values, O_2_ consumption per mole of active material (Fig. 2c) can be quantified from:

$$O_{2,cons}=\frac{Ak_{L}\left( C_{O2,int}-\left[ O_{2} \right] \right)}{V_{tot}} (S20)$$

where *V*_tot_ is total basis concentration of active material.

**Optimal system scaling in air**

From:

$$\frac{d[O_{2}]}{dt}+\left[ O_{2} \right]\left( \frac{Ak_{L}+S_{A}k_{dir,O2}+Vk_{O2, V+}\left[ V^{+\bullet} \right]+Vk_{O2, V0}\left[ V^{0} \right]}{V} \right)= \frac{Ak_{L}C_{O2,int}}{V} (S18)$$

and:

$$SOC=\frac{It}{C_{bat}} (S19)$$

(S19) can be substituted into (S18) and both sides can be divided by *C*_O2,int_ to give the dimensionless equation:

$$\frac{dx_{O2}}{dSOC}+x_{O2}\left( \frac{Ak_{L}+S_{A}k_{dir,O2}+Vk_{O2, V+}\left[ V^{+\bullet} \right]+Vk_{O2, V0}\left[ V^{0} \right]}{V} \right)\left( \frac{C_{bat}}{I} \right)= \frac{C_{bat}Ak_{L}}{IV} (S21)$$

where:

$$x_{O2}=\frac{\left[ O_{2} \right]}{C_{O2,int}} (S22)$$

From the equation above, 4 independent dimensionless groups emerge that serve as dimensionless time constants for each of the processes that either generate or consume O_2_:

$$\left( \frac{C_{bat}Ak_{L}}{IV} \right), \left( \frac{{C_{bat}S}_{A}k_{dir,O2}}{IV} \right), \left( \frac{C_{bat}Vk_{O2, V+}\left[ V^{+\bullet} \right]}{IV} \right),\left( \frac{C_{bat}Vk_{O2, V0}\left[ V^{0} \right]}{IV} \right)$$

From the equation above, 4 independent dimensionless groups emerge that serve as independent time constants for each of the processes that either generate or consume O_2_. As there are three processes that consume O_2_ and one process that generates it, the time constant for O_2_ generation serves as a natural normalisation factor to give insights into the scale-independent relative rates of consumption to generation. Thus, normalising, we get the following three dimensionless groups:

$${Da}_{II elec}= \frac{{S_{A}k}_{dir,O2}}{{Ak}_{L}} {Da}_{II V+\cdot}= \frac{{Vk}_{O2, V+}\left[ V^{+\bullet} \right]}{{Ak}_{L}} {Da}_{II V0}= \frac{{Vk}_{O2, V0}\left[ V^{0} \right]}{{Ak}_{L}}$$

As in these groups, time constants associated with (electro)chemical processes that consume O_2_ are compared against the rate of diffusive mass transfer and equilibrium partitioning of O_2_ into bulk solution, these dimensionless quantities represent second Damköhler numbers (*Da*_II_) – a class of dimensionless group commonly used in chemical engineering to describe the rate of chemcial reaction to the rate of diffusive mass transfer.^8^

For a cylindrical tank of constant apsect ratio 1, which couples the scaling of *A* to *V*, *Da*_II_ for each O_2_ consumption process can be varied with scale for different values of electrode area (*S_A_*), which scale independently.

Using respective *k* values for methyl viologen in Table S1 and using maximum radical concentration to determine *Da*_II V+_, the sensitivity of *Da*_II V+_ to *K_c_* and *K_d_* can be evaluated as show below.

**Fig. S3 | Sensitivity of *Da*_II V+_ to thermodynamic parameters. (a)** Second Damköhler number *vs.* electrolyte volume when *K_c_* is varied by ten orders of magnitude when *K_d_* = 1. **(b)** Second Damköhler number *vs.* electrolyte volume when *K_d_* is varied by three orders of magnitude when *K_c_* = 10^10^.

When *K_c_* = 1 and *K_d_* = 100 and *k* is varied by one order of magnitude from its value for methyl viologen (Table S1), the sensitivity of *Da*_II V+_ to *k* can be evaluated as show below.

**Fig. S4 | Sensitivity of *Da*_II V+_ to kinetic parameters.** Second Damköhler number *vs.* electrolyte volume when *K_c_* = 1, *K_d_* = 100 and *k* is varied by one order of magnitude.

**S3. EPR and UV-Vis data**

*EPR data*

EPR radical concentration data for methyl viologen are as previously reported.^4^ Briefly, radical concentration (*C*_s_) was quantified by spin counting, that is, taking the double integral (DI) of the EPR signal according to:

$$DI=C_{ns}\frac{{n_{B}QB}_{m}\sqrt{P}S(S+1)C_{s}v}{f(B_{1}, B_{m})}$$

where *C*_ns_ is a constant including the normalised spectrometer settings, *i.e.* sweep time and number of accumulations, *P* is the microwave power, *B*_m_ is the modulation amplitude, *Q* is the resonator’s quality factor, *n*_B_ is the Boltzmann factor for temperature dependence, *S* is the total electron spin, *v* is the volume of the sample, and *f*(*B*_1_,*B*_m_) is the spatial distribution of microwave and modulation field in the sample. For the data shown in Fig. 1a, a methyl viologen basis concentration of 10 mM was used while a full cell was cycled for a full charge discharge cycle at a constant current of 10 mA. Voltage cut-offs of 0.5 V and 2.05 V were used.

*UV-Vis data*

UV-Vis concentration data for methyl viologen are as previously reported.^4^ Briefly, a UV-Vis spectrometer is setup to have an electrolyte flowing through it during electrochemical cycling. Thus, there is a time series of UV-Vis spectra which changes over time.

Beer-Lampert law connects the absorption to a concentration, at a wavelength, $\lambda$:

$$A\left( \lambda\right)=\epsilon\left( \lambda\right)*l*c$$

For a fixed setup, where only the concentration, *c*, is changing, as all the values of $\lambda$ are explored. So, we can get a value for the molar extinction coefficient, $\epsilon$ at every wavelength. Note: we assume linearities here, which will not hold true for very concentrated/absorbant systems, as the absorbance, *A*, is not reliably linear above values $\geq2$.

By assuming linear behaviour throughout, we can split the overall *in situ* UV-Vis spectra into its components, as each molecule has a distinct UV/Vis spectra. Thus, in our case (for the cycling of MV), we have

$$A_{total}=A_{MV^{2+}}+A_{MV^{+\bullet}}+A_{MV^{0}}+A_{MV^{+\bullet}\cdots MV^{+\bullet}}$$

As the pure spectra of each component is stable over time, just their concentration varies:

$$A_{total}\left( t \right)=\epsilon_{MV^{2+}}*l*c_{MV^{2+}}\left( t \right)+\epsilon_{MV^{+\bullet}}*l*c_{MV^{+\bullet}}\left( t \right)+\epsilon_{MV^{0}}*l*c_{MV^{0}}\left( t \right)$$

$$+\epsilon_{MV^{+\bullet}\cdots MV^{+\bullet}}*l*c_{MV^{+\bullet}\cdots MV^{+\bullet}}\left( t \right)$$

As the measured UV-Vis data are the full time series of $A_{total}\left( t \right)$, we can use that to decompose the overall spectra into its components using non-negative matrix factorisation (NNMF). NNMF breaks the time series/data matrix into two matrices that when multiplied together result in the data matrix (*i.e.*, pure spectra and their concentrations over time). So, the results from NNMF are a matrix of pure spectra (the $l$ can be considered as a pre-factor, ignored for this demonstration):

$$\left[ \begin{matrix} \cdots\epsilon_{MV^{2+}}\left( \lambda\right)\cdots\\ \cdots\epsilon_{MV^{+\bullet}}\left( \lambda\right)\cdots\\ \cdots\epsilon_{MV^{0}}\left( \lambda\right)\cdots\cdots\epsilon_{MV^{+\bullet}\left( \lambda\right)\cdots MV^{+\bullet}}\left( \lambda\right)\cdots\end{matrix} \right]$$

and then a time series:

$$\left[ \begin{matrix} c_{MV^{2+}}\left( 0 \right) & c_{MV^{+\bullet}}\left( 0 \right) & c_{MV^{0}}\left( 0 \right) & c_{MV^{+\bullet}\cdots MV^{+\bullet}}\left( 0 \right) \\ c_{MV^{2+}}\left( 1 \right) & c_{MV^{+\bullet}}\left( 1 \right) & c_{MV^{0}}\left( 1 \right) & c_{MV^{+\bullet}\cdots MV^{+\bullet}}\left( 1 \right) \\ \vdots& \vdots& \vdots& \vdots\\ c_{MV^{2+}}\left( t \right) & c_{MV^{+\bullet}}\left( t \right) & c_{MV^{0}}\left( t \right) & c_{MV^{+\bullet}\cdots MV^{+\bullet}}\left( t \right) \end{matrix} \right]$$

These two multiplied together would then result in the original spectra.

The underlying data were then analysed with both the NNMF and Principal component analysis (PCA) algorithms, as implemented in the sklearn Python package. The final components were assigned as: MV^+•^, MV^0^*_(s)_*, MV^2+^*_(aq)_*, and π-(MV*^+^*^•^)_2_ _(_*_aq)_*_._ These component for MV^2+^*_(aq)_* was then rescaled by assuming an initial concentration of 10 mM, as implemented experimentally.

**S4. References**

[1] *Nature* **579**, 224-228 (2020).

[2] *J. Am. Chem. Soc.* **143**, 1885-1895 (2021).

[3] *Nature* **623**, 949-955 (2023).

[4] Carrington, M.E. PhD Diss. *University of Cambridge* (2024).

[5] *Biochim. Biophys. Acta - Bioenerg.* **333**, 487-496 (1974)

[6] *J. Electroanal. Chem.* **799**, 53-60 (2017).

[7] *Environ. Sci. Pollut. Res. Int.* **12**, 66-70 (2005).

[8] *Z. Elektrochem. Angew.* *Phys. Chem.* **42**, 846-862 (1936).
